# Developing a Discrete Choice Experiment Instrument for Evaluating Patients’ Preferences in Precision Oncology

**DOI:** 10.5812/ijpr-141797

**Published:** 2024-08-27

**Authors:** Zahra Karimi Majd, Nazila Yousefi, Mohammad Peikanpour, Mohammad Sistanizad, Ghader Mohammadnezhad, Behniya Azadmehr, Farzad Peiravian

**Affiliations:** 1 Department of Pharmacoeconomics and Pharma Management, School of Pharmacy, Shahid Beheshti University of Medical Sciences, Tehran, Iran; 2 Department of Clinical Pharmacy, School of Pharmacy, Shahid Beheshti University of Medical Sciences, Tehran, Iran; 3 School of Pharmacy, Shahid Beheshti University of Medical Sciences, Tehran, Iran

**Keywords:** Cancer, Patients’ Preferences, Discrete Choice Experiment, Precision Oncology, Precision Medicine, Preference Measurement Tool

## Abstract

**Background:**

In addition to clinical and technical considerations, patients’ preferences are essential for evaluating interventions such as precision medicine (PM).

**Objectives:**

This study aimed to identify and prioritize attributes of precision oncology that are important for patients to develop and validate a standard stated preference instrument.

**Methods:**

The key attributes of precision oncology and their related levels were extracted from the systematic literature review and were presented on a validated 5-point Likert scale questionnaire to experts (n = 35). In two rounds of Delphi, participants scored and prioritized the attributes through this personally administered questionnaire to identify the five most important ones to develop a discrete choice experiment (DCE) instrument. The developed DCE questionnaire was subsequently validated, providing a robust and standard instrument for evaluating patients’ preferences for precision oncology.

**Results:**

Based on the consensus criteria, the final DCE included four attributes and a total of 14 levels, which were access to treatment (easy/not easy), out-of-pocket (OOP) expenditures (four levels according to treatment costs in the country), change in life expectancy (LE, six levels from an average gain of three months to four years), and change in quality of life (QoL, improvement or no change).

**Conclusions:**

The above-mentioned attributes represent patients’ main preferences from the views of the Iranian experts. The developed DCE questionnaire can be used to assess patients’ preferences and willingness to pay (WTP) in precision oncology.

## 1. Background

In recent years, it has been increasingly evident that cancer is a complex disease with varying responses to generic treatments, such as chemotherapy and radiation (1). The “one size fits all” approach to cancer therapy is too simplistic and often results in ineffective, expensive treatments with unnecessary toxic adverse effects and costs for patients (2). Oncologists consider not only the genetics and biology of cancer but also the age, medical condition, lifestyle, and goals of each patient when deciding on treatment strategy. The precision medicine (PM) approach, which tailors treatments to the specific tissue, gene mutations, and personal factors relevant to each unique case of cancer, has improved patient health in recent years. For instance, in the case of human epidermal growth factor receptor 2 (HER2)-positive breast cancer, trastuzumab has emerged as a paradigm-shifting therapeutic agent that illustrates the power of PM in oncology (3).

Patient centricity is characterized by respecting and responding to individual patient preferences, needs, and values to optimize the information and achieve the most beneficial patient outcomes (4). Patients’ preferences are progressively emerging as essential in health decision-making and drug development, reflecting the shift from a “physician-dominated decision-making” to a more active and decisive role for patients (5). The valuation of the interventions through patients’ preference data is crucial to facilitate the successful implementation of precision oncology, as acknowledged by organizations such as the European Network for Health Technology Assessment (EUnetHTA) (6). Such data can help estimate their willingness to pay (WTP) for treatments, predict the market diffusion of various PM outcomes, and determine how demand will be affected by factors such as health technology prices and evolving evidence (7).

Moreover, obtaining consumer-centric information can aid stakeholders in anticipating behaviors regarding implementing new care paradigms, such as PM (8); nonetheless, no standard instrument to measure it can be found in the literature. Discrete choice experiment (DCE) is one of the widely used instruments for quantifying stated preferences for health. This method is rooted in Random Utility Theory, which assumes that treatments can be described by specific attributes, such as the likelihood of a positive test outcome. Individuals’ preferences are determined based on the levels of these attributes, such as different probabilities. Respondents are presented with hypothetical scenarios that vary these attribute levels and are asked to choose their preferred scenario in each choice task (9).

## 2. Objectives

This study aimed to seek consensus from experts in Iran regarding the attributes of precision oncology to inform subsequent research and develop a standard and valid DCE instrument to measure patient preferences, as researchers developing a DCE are tasked with creating a limited set of attributes based on a rigorous process, including a literature review and expert opinions to make sure the DCE instrument is standard and not overly complex or burdensome.

## 3. Methods

### 3.1. Ethics Statement

This study received ethical approval (ID: IR.SBMU.PHARMACY.REC.1400.174) from the Research Ethics Committee of the School of Pharmacy at Shahid Beheshti University of Medical Sciences in Tehran, Iran.

### 3.2. Systematic Search

The Preferred Reporting Items for Systematic Reviews and Meta-analyses (PRISMA) guideline (10) was followed to systematically review the studies on patients’ preferences in precision oncology and genetic tests. Search terms were: Patient* preference* OR attribute* OR discrete choice experiment AND precision medicine OR personalized medicine AND cancer OR neoplasm*. Multiple databases were included in the systematic search strategy, namely PubMed, ScienceDirect, and Cochrane Library. The full search strategy for each database is presented in “Appendix 1: Search Strategy”. The references obtained by searching through databases were imported to Mendeley (a reference manager software), and duplicates were removed. Studies were screened for eligibility based on the title and abstract, followed by full-text screening by two researchers. The search was conducted in February 2022 and was restricted to peer-reviewed journals published in English. The included articles’ reference list was checked to find additional relevant publications. No restrictions were placed on the time of publication. Two researchers extracted and summarized the attributes to be presented on a 5-point Likert scale structured questionnaire to experts through a Delphi technique.

### 3.3. Participants and Recruitment

To ensure a diverse range of expertise among the participants, it was aimed to recruit at least 30 experts via maximum variation purposeful and snowball sampling techniques, including clinical oncologists, clinical pharmacists, government officials, healthcare payer specialists, and pharmacoeconomists (11). The definition of a national expert is an individual who meets the following inclusion criteria:

1- having work experience in a senior professional title and 2- engaging in health decision-making

### 3.4. Data Collection

The Delphi technique is a widely accepted systematic approach in healthcare to establish a consensus from respondents within a specific domain of expertise (12). The participants responded to the structured, personally administered questionnaires through a modified Delphi approach. One of the modifications was utilizing a structured questionnaire for the rounds, and another was reaching consensus in limited rounds (Table 1). A significant body of research supports both of these modifications (13). They rated and prioritized the attributes on a 5-point Likert scale. This modified Delphi approach involved two rounds of consensus. In the first round, the panelists received a thorough introduction to state the research inquiry, the study process, definitions of the candidate attributes and their levels, the previous steps taken to extract them from the literature, and the criteria set forth for the inclusion of attributes in subsequent rounds. They were then required to rate their level of agreement with each attribute on a 5-point Likert scale (5 = Definitely include, 4 = Possibly include, 3 = Neutral, 2 = Possibly exclude, 1 = Definitely exclude). In the first round, at the end of the questionnaire, an open question was put for the experts to add if they think of other attributes. To enable a better comprehension of the expert opinion, a five-point scale was implemented instead of a binary response, enabling the participants to express their degree of agreement with the inclusion, whether stronger or weaker (14).

**Table 1.** Final Discrete Choice Experiment Attributes and Their Levels That Reached Consensus

Attribute	Levels
**Access to treatment**	Easy/not easy
**Change in quality of life**	Improvement/no change
**Change in life expectancy**	Between a gain of 1 month to a gain of 5 months, average gain of 3 months
	Between a loss of 3 months to a gain of 9 months, average gain of 6 months
	Between a gain of 6 months to a gain of 1.5 years, average gain of 1 year
	Between a loss of 6 months to a gain of 2.5 years, average gain of 1 year
	Between a gain of 0 years and a gain of 8 years, average gain of 4 years
	Between a gain of 2 years and a gain of 6 years, average gain of 4 years
**Out-of-pocket expenditures in 6 months**	Four levels based on comparable treatment expenditures

### 3.5. Data Analysis

Descriptive statistics were used to analyze the results of each round of the modified Delphi method. The goal was to identify the maximum five most important attributes to serve as a limited set of candidate attributes for inclusion in a DCE and prevent complexity and bothersomeness for the participants. The literature emphasizes the significance of defining consensus prior to initiating the first Delphi round. The consensus criteria were defined as a mode value of 5, a standard deviation (SD) of < 1, and an over 85% agreement in the total score of 4 and 5. The study team blindly evaluated the attributes’ scores to reduce the error rate. The mode score, SD, and sum of the agreement scores 4 and 5 were calculated for each attribute. The ones meeting the consensus criteria were included in the next round for further narrowing down and refinement. For the subsequent round of Delphi, all modifications were summarized and presented on a Likert scale, and the experts scored and prioritized the revised attribute list to achieve consensus. Attributes achieving consensus were included in the final DCE instrument. Finally, the face and content validity of the developed DCE instrument was assessed by a group of 12 experts independent of the instrument’s development process, by clarity and ease of understanding, and by calculating the CVI and CVR to develop a standard and validated DCE questionnaire for further research.

## 4. Results

**Figure 1.**
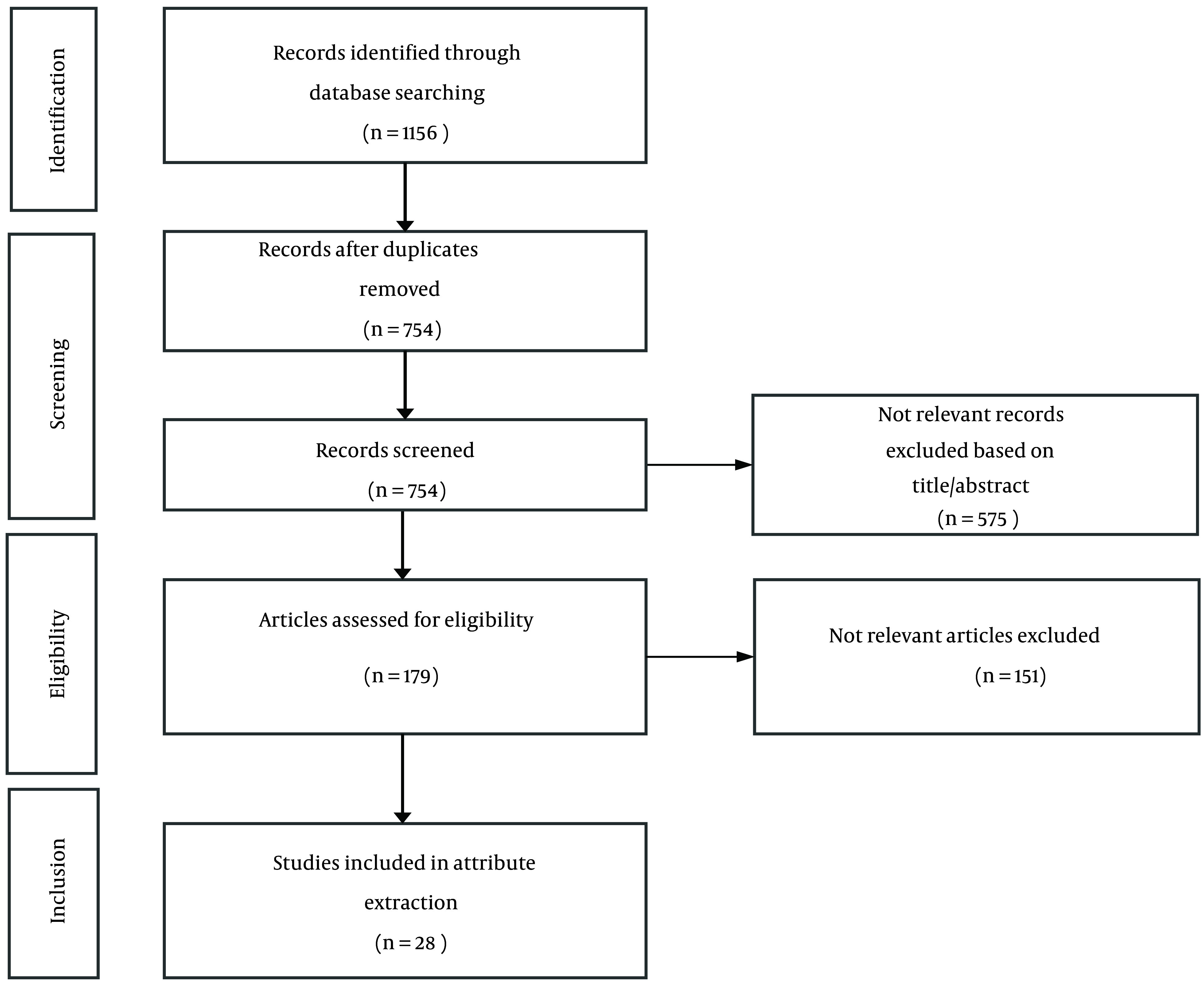
PRISMA flow diagram for study selection

The present systematic review identified 1,156 records. Following the screening and eligibility evaluation, 28 studies were deemed eligible and were subsequently included in the review and attribute extraction (Figure 1). Seven potential attributes extracted from the selected studies included health provider’s recommendation (yes/no), treatment frequency/duration (frequency: Every month/every two weeks, duration: From a few weeks to a few years), predictability and efficacy (high/medium/low), out-of-pocket (OOP) expenditures (four levels based on treatment expenditures), adverse events (the type and severity of adverse effects), change in life expectancy (LE, six levels from an average gain of five months to four years), and change in the quality of life (QoL, improvement/no change) (Table 2). 

**Table 2.** Extracted Attributes from the Literature and Their Citations

Attribute	Citations
**1. Doctor’s recommendation**	(15), (16-19)
**2. OOP expenditures**	(16-20), (21-24), (25-27)
**3. Treatment frequency/duration**	(21, 22), (27-30), (31-33)
**4. Adverse effects**	(21-24), (26-30), (31-35), (36-39)
**5. Change in QoL**	(16, 17), (22), (24), (26), (34), (36), (38)
**6. Predictability and efficacy**	(8), (15), (16-18), (20), (27)
**7. Increase in LE**	(8), (16, 17), (19-24), (26-30), (31-33), (35-37), (39, 40)
**8. Familial benefits ** ^**a**^	
**9. Reimbursement status ** ^**a**^	
**10. Access to treatment ** ^**a**^	

Abbreviations: LE, life expectancy; OOP, out-of-pocket; and QoL, quality of life.

^a^ These attributes were added by the experts during the first Delphi round; therefore, the experts scored and prioritized these attributes at the end of the first Delphi round as a supplementary question.

These attributes and their levels were presented on a validated 5-point Likert scale, personally administered structured questionnaire to the experts through a Delphi process for scoring and prioritizing. This questionnaire’s validation process included content validity assessments that were carried out by calculating the CVI and CVR. All the attributes and their levels were deemed acceptable and necessary. Table 3 shows all the attributes, their levels, and a short description that was presented to the participants in the questionnaire in Delphi rounds.

**Table 3.** Original 5-Point Likert Scale Personally Administered Questionnaire Used in the Delphi Rounds

Attribute	Description	Levels	More Importance from Left to Right				
			1	2	3	4	5
**1. Doctor’s recommendation**	If the doctor/oncologist recommends precision medicine to the patient	Yes/no					
**2. Out-of-pocket expenditures**	The amount that the patient pays out of pocket	Four levels based on treatment expenditures					
**3. Reimbursement status ** ^**a**^	If the insurance covers the treatment	Yes/no					
**4. Treatment duration/frequency**	The time it takes for the treatment to be complete	From a few weeks to a few years					
**5. Adverse effects**	The adverse events of the treatment	Type and severity of adverse effects					
**6. Quality of life gains**	Function of the patient and the ability to do daily activities	Improvement/no change					
**7. Predictability and efficacy**	The degree of certainty of the association of biomarker or gene mutation with the clinical outcome of treatment and its effectiveness	High/medium/low					
**8. Life expectancy change**	Change in patient’s life expectancy after treatment	From a few months to a few years ^b^					
**9. Familial benefits ** ^**a**^	Awareness in case of family illness	Yes/no					
**10. Access to treatment ** ^**a**^	If the treatment is easily accessible	Easy/not easy					

^a^ These attributes were suggested by the study participants, and they were scored at the end of the first Delphi round in a supplementary question on a 5-point Likert scale and again in the second Delphi round, among other attributes.

^b^ Between a gain of 1 month to a gain of 5 months, an average gain of 3 months/Between a loss of 3 months to a gain of 9 months, an average gain of 6 months/Between a gain of 6 months to a gain of 1.5 years, an average gain of 1 year/between a loss of 6 months to a gain of 2.5 years, an average gain of 1 year/Between a gain of 0 years and a gain of 8 years, an average gain of 4 years/Between a gain of 2 years and a gain of 6 years, an average gain of 4 years.

A total of 35 national experts who met the eligibility criteria were recruited for the present study, comprising specialists in five different fields related to the study’s aim, including oncologists and clinical pharmacists (n = 10), government officials (n = 8), healthcare payer specialists (n = 6), and pharmacoeconomists (n = 11). The average years of practice were 16 (SD = 6.5). Most experts (n = 31) had senior professional titles and were highly engaged in health management. The median length of the Delphi rounds was about 16 minutes. Anonymity was maintained throughout the Delphi rounds.

**Figure 2.**
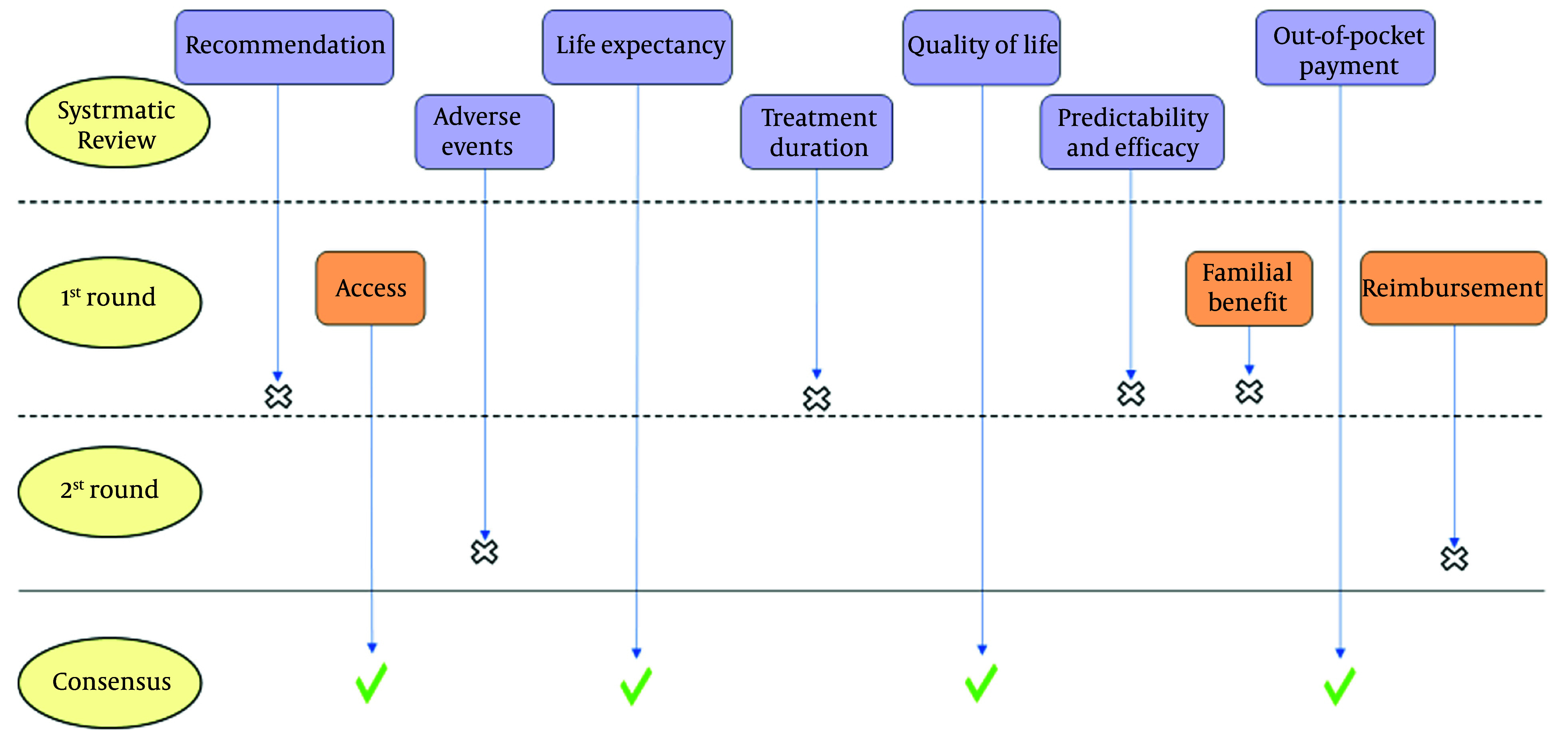
Flowchart of the Delphi process

Figure 2 illustrates a schematic flowchart of the Delphi process. During the first Delphi round, three additional attributes, including reimbursement status (yes/no), familial benefits (yes/no), and access to treatment (easy/not easy), were added by the experts. The experts highlighted the importance of patients’ access to treatment as a critical issue in Iran. According to their experience, sometimes the preferred treatments in the guidelines could not be provided to the patient due to the lack of access due to different causes. Reimbursement status is another important characteristic that the present study’s clinical experts highlighted its importance. A few participants also pointed out the familial benefit, as the genetic test is one of the first steps of precision treatment, which can predict whether the cancer is familial. Therefore, all of the present study’s participants scored these additional attributes at the end of the first Delphi round as a supplementary question on a 5-point Likert scale and in the second round among other attributes.

At the end of the first round of the modified Delphi method, four attributes (doctor’s recommendation, predictability and efficacy of the treatment, treatment frequency/duration, and familial benefits) were eliminated based on not meeting the consensus criteria which were agreed upon beforehand as a mode score value of 5, an SD of < 1, and an over 85% agreement in the total scores of 4 and 5.

After integrating the results from the initial round into the remaining six attributes, the refined survey questionnaire was administered for a second round. At the end of the second round, two attributes (reimbursement status and adverse events) were removed based on not meeting the consensus criteria. By the end of the second round, the present study’s objective of obtaining consensus about attributes was achieved to develop a standard DCE instrument that is not complex and easy to understand for the patients. Table 4 shows the quantitative results of the Delphi phases.

**Table 4.** Results of the Delphi Phases ^a^

Attributes	First Delphi Cycle					Second Delphi Cycle				
	Mode Score	SD	Definitely Include ^b^	Possibly Include ^c^	SUM 5 + 4 ^d^	Mode Score	SD	Definitely Include ^b^	Possibly Include ^c^	SUM 5 + 4 ^d^
**1. Doctor’s recommendation**	3	1	25.7	25.7	51.4	-	-	-	-	-
**2. Access ** ^**e**^	5	0.45	85.7	11.4	97.1	5	0.46	71.4	28.6	100
**3. OOP expenditures**	5	0.53	71.4	25.7	97.1	5	0.51	77.1	20	97.1
**4. Reimbursement ** ^**e**^	5	0.73	51.4	34.3	85.7	4	0.91	28.6	51.4	80.0
**5. Treatment duration/frequency**	2	0.66	45.7	0.0	45.7	-	-	-	-	-
**6. Adverse events**	5	0.71	42.9	42.9	85.7	4	0.85	25.7	37.1	62.9
**7. QoL**	5	0.66	60.0	37.1	97.1	5	0.43	77.1	22.9	100
**8. Predictability and efficacy**	4	0.84	45.7	40.0	85.7	-	-	-	-	-
**9. LE**	5	0.48	65.7	34.3	100	5	0.38	82.9	17.1	100
**10. Familial benefit ** ^**e**^	2	1.3	20.0	28.6	48.6	-	-	-	-	-

Abbreviations: LE, life expectancy; OOP, out-of-pocket; QoL, quality of life; and SD, standard deviation.

^a^ Values are expressed as No. (%) unless otherwise indicated.

^b^ Definitely include: The number of times the attribute received a score of 5 on the Likert scale in percentage.

^c^ Possibly include: The number of times the attribute received a score of 4 on the Likert scale in percentage.

^d^ SUM 5+4: The total number of times the attribute received a score of 4 and 5 on the Likert scale.

^e^ Additional attributes that were added by our experts in the first Delphi round.

Of all the ten attributes posed, four attributes, including OOP payments of the treatment (four levels based on treatment expenditures), access to treatment (two levels, including easy and not easy), QoL (two levels, including improvement and no change), and LE after treatment (six levels from an average gain of five months to four years), met the consensus criteria and were included in the attributes’ list for developing the DCE instrument. In order to evaluate the DCE instrument, face, and content validity assessments were carried out. The final generated DCE instrument consisted of a list of four attributes and a total of 14 levels. The complete DCE questionnaire was developed via JMP software in four surveys with three choice tasks each and can be found in Appendix 2 (Table 5). Table 6 shows the results of the validity calculations.

**Table 5.** An Example of a Random Choice Task

	If You Have 2 Options to Choose from for the Cancer Treatment, Which one Will You Prefer? (Please, Tick the Below Box)	
Treatment characteristics	Option A	Option B
**Access to treatment**	Not easy	Easy
**Change in quality of life**	Improvement	No change
**Effect on life expectancy**	Between a gain of 2 years and a gain of 6 years, an average gain of 4 years	Between a loss of 6 months to a gain of 2.5 years, an average gain of 1 year
**Out-of-pocket expenditures in 6 months**	$1,600	$1,000
	Prefer treatment option A □	Prefer treatment option B □

**Table 6.** Final Discrete Choice Experiment Questionnaire’s Face and Content Validation Results (Ratings of the Items by 12 Experts: Items Rated 3 or 4 on a 4‑Point Relevance Scale)

Attributes	CVR	Face Validity	I-CVIs	S-CVI/UA ^a^	S-CVI/Ave ^b^
**Access to treatment**	1	3.5	0.94	S-CVI = 0.94; S-CVI/UA = 0.49	0.983
**OOP payments**	0.96	3.5	1		
**Life expectancy**	1	3.5	1		
**Quality of life**	0.986	3.3	0.993		

Abbreviations: CVR, contingent value right; CVI, content validity index; I-CVIs, item-CVI; S-CVI/UA, scale-CVI based on the universal agreement method; S-CVI/Ave, scale-CVI based on the average method; and OOP, out-of-pocket.

^a^ S-CVI/UA: The proportion of items on a scale that achieves a relevance rating of 3 or 4 by all the experts.

^b^ S-CVI/Ave: Average of the I-CVIs for all items on the scale.

## 5. Discussion

Assessing the value of interventions and investigating attributes that influence patients’ preferences toward precision oncology and derived WTP estimates in hypothetical treatment options is important to enabling the effective implementation of precision oncology and ensuring that public health spending is allocated toward the most effective strategies that are preferred by the consumers and can benefit the health of the population as a whole. Although some studies have assessed attributes in oncology, there is a gap in the literature regarding using a standard and validated DCE instrument to evaluate these attributes and patients’ WTP in the context of precision oncology. This gap is significant as precision oncology becomes more widely used in clinical practice, and patient preferences are critical for shared decision-making. Therefore, this study contributes to the literature by developing a DCE instrument explicitly tailored to the attributes of precision oncology treatment concerning the “average” patient with cancer. This instrument can be valuable in measuring patients’ preferences and implementing precision oncology programs that align with patients’ needs, preferences, and WTP. Additionally, since work has yet to be performed on this matter in Iran, patients as one of the stakeholders can be used in pricing, determining the service tariff, and determining the reimbursement program. In the following section, the implications of different attributes employed in this study are discussed.

The findings of the present work confirm that in the delivery of healthcare services, change in QoL and future life expectations are key attributes, as is proven in several studies for cancer patients, especially in older patients with naturally limited LE (41-43). It has been revealed in previous studies that patients can have equal preferences for QoL and LE or prefer each of them more, and the preference is associated with different factors, such as age, gender, educational attainment, and the nature of their oncologist communication. The current study’s results revealed that healthcare professionals’ disciplines influence the preferences for change in LE and QoL among patients. Specialists and clinicians value LE more than the QoL; however, healthcare payer (insurance) specialists were more concerned about the QoL than LE. These attributes held significant value and were ultimately incorporated into the final DCE instrument.

The results of this study suggest that OOP payments are more important to patients than whether the treatment is covered by insurance (reimbursement status), a finding consistent with several studies of other cancer treatments or diagnoses (44). However, health providers emphasized that precision treatment must be as insured as conventional treatments, as it is a crucial attribute in their point of view. Still, considering that the financial burden of treatment can be graded according to OOP payments and health insurance status, other professional groups believed that the OOP payments were more important to the patient and reimbursement status does not automatically translate into financial protection from medical-related expenditures. Including the OOP payments attribute in a DCE survey allows an estimation of marginal WTP for each service attribute and its levels. As the cost has been one of the crucial attributes of the treatment for patients and healthcare payers, using precision treatment and performing more tests to diagnose a disease increases healthcare expenditures. However, with the disease’s high psychological burden for the cancer patient, precision treatment can be very beneficial, with maximal response to treatment and minimal adverse events. Therefore, PM will benefit insurance companies in the long term because information about a person’s disease and responsiveness to different interventions and treatments will help develop disease-prevention approaches (45).

Another attribute of PM is clinical consensus (i.e., doctor’s recommendation). Although some studies show that this attribute is critical from the perspective of payers (46), several studies showed that cancer patients are willing to accept and pay for PM when such treatment has been deemed clinically useful by their healthcare provider (47-49). The current study’s results showed that, on the one hand, for a treatment to be covered by insurance, clinical consensus upon the treatment is necessary. On the other hand, this attribute is critical for cancer patients with PM in their disease trajectory, and PM is not an option for them if their healthcare provider does not recommend it.

Additionally, the access to treatment attribute, which holds significant importance in Iran, was introduced as a salient attribute despite its limited consideration in the existing literature. Ensuring access to treatments, especially innovative technologies, is crucial to the implementation and uptake of PM. After introducing treatment to patients, healthcare professionals in the present study attached high importance to this attribute for several reasons. Sanctions have negatively impacted access to medical technologies, such as diagnostic kits, genetic databases, and drugs, particularly those that rely on importing raw materials or finished products (50); the implementation of PM as an innovative technology could result in unequal access and prevent some patients from experiencing potential benefits (15). In order to overcome these challenges, policymakers need to adopt a systematic and coordinated strategy to ensure nationwide market accessibility, thereby mitigating the impact of limited access on patients’ ability to obtain recommended treatments (51).

Similarly, access to precision oncology is crucial for improving cancer treatment outcomes and reducing health disparities. Precision oncology, which uses genomic information to guide the selection of targeted therapies for individual patients, has revolutionized cancer diagnosis and treatment. However, access to precision oncology is only sometimes universal. It varies based on socioeconomic status and location, resulting in some patients needing access to the latest treatments and technologies and receiving less effective standard care. Improving access to precision oncology is vital for more accurate diagnoses, personalized and effective treatment plans, and improved patient outcomes. To address these disparities, investment in the development and dissemination of precision oncology tools and technologies, in addition to the education and training of healthcare providers, is necessary to ensure that all patients have access to the most advanced treatments available (52, 53).

To subsequently discuss the further attributes evaluated in this study, the predictability and efficacy of the treatment are of utmost importance to patients. The advancement of PM is crucial for providing optimal cancer care. By enhancing predictability and efficacy, precision oncology holds the potential to enhance the accuracy of diagnoses, personalize treatment plans, and ultimately improve patient outcomes and QoL. However, significant amounts of data are required to support the estimates of accuracy and clinical utility, as noted in the literature (7). At the same time, the recommendation for cancer treatment by healthcare providers typically takes into account the stage of the disease, the patient’s health, and the availability of treatments. The present study’s experts believe that a healthcare provider’s recommendation can give patients confidence in prioritizing targeted treatment.

Another attribute evaluated in the present study is the adverse effects of the cancer treatment. Garfeld et al. have observed that consumers value PM outcomes more than their adverse effects. In the latter study, most individuals (77%) would prefer a cancer treatment with a higher chance of success, even if it had higher side effect rates, compared to a treatment with a lower chance of success but fewer side effects (23%). Likewise, the experts in the current study determined that this attribute could not be among the most critical attributes from the patient’s perspective (8).

This study has several implications. The viewpoint of both healthcare providers and payers will assist in aligning the development programs of tests and therapeutic products with decision-makers. Furthermore, the differing perspectives on the attributes of PM gathered in this study can guide the creation of value-based evaluations by the industry, payers, and healthcare providers. The present study’s findings could enhance HTA activities by highlighting critical areas for evidence collection, generation, and presentation aligned with patient preference. Experts’ points of view on disease attributes can be used to make predictions about PM uptake, which can be helpful for drug developers concerning market planning, pricing, and evidence generation. Payers can further use them to inform financial planning and future budget impact estimates. In this study, the experts in the health system field were chosen to prioritize precision oncology treatment attributes. Their expertise and objective evaluation of treatment options make their input important in decision-making.

This study is relevant to the current trend of a growing demand for patient-centered healthcare services and increased emphasis on patient choice (54, 55).

Although this study was carefully designed and satisfactory results from validity tests were obtained, it is important to recognize the following limitations of the study. One of the limitations of the Delphi method is that it involves limited statistical analysis; instead, the results are based on the collective judgments and opinions of the participants, which can be influenced by individual biases or preferences. Additionally, the Delphi method is often used in cases where there is limited existing knowledge or data on a topic. Although this can be an advantage, it can also limit the ability to explore the range of possible outcomes or solutions.

### 5.1. Conclusions

In this paper, using a systematic review and the Delphi method to identify and prioritize precision oncology treatment characteristics, a DCE instrument has been developed to measure patient preferences toward precision oncology. The novelty of the work relies on the opinion of experts who objectively assess treatment options. The experts articulate and prioritize the attributes of precision oncology with their comprehensive perspectives; this could indicate further future exploitation of patients’ preferences and lay the foundation for PM in chronic diseases, such as cancer. In this instrument, using the DCE method, four characteristics of access to treatment, OOP payments, QoL, and LE with different levels are included. This has the potential to enhance patient health by addressing some of the obstacles faced by PM use in clinical practice.

## supplementary material

ijpr-22-1-141797-s001.pdf

## Data Availability

The dataset presented in the study is available on request from the corresponding author during submission or after publication.
