# Supplementary material for: Developing a Discrete Choice Experiment Instrument for Evaluating Patients’ Preferences in Precision Oncology
Source: Iran J Pharm Res. 2024 Aug 27;22(1):e141797. doi: 10.5812/ijpr-141797 (PMC12523849; doi:10.5812/ijpr-141797)
Supplement: ijpr-22-1-141797-s001.pdf [file ijpr-22-1-141797-s001.pdf]

**Iran J Pharm Res.:e141797.**

**https://doi.org/10.5812/ijpr-141797**

**SUPPLEMENTARY MATERIALS**

**Appendix 1 – SEARCH STRATEGY**

| Database | Search Strategy | Results |
|---|---|---|
| PubMed | ("Patient Preference"[Mesh] OR "Sociological Factors"[Mesh] OR "Disease Attributes"[Mesh]) AND "Precision Medicine"[Mesh] AND "Neoplasms"[Mesh] NOT "Sequence Analysis, DNA"[Mesh] NOT "Next-Generation Sequencing" | 919 |
| Science Direct | title-abs-key ((Preference OR Attribute OR discrete choice experiment) AND (Precision Medicine OR personalized medicine) AND (Neoplasm OR cancer)) | 214 |
| Cochrane Library | ID Search<br>#1 ("preference" OR "attribute"):ti,ab,kw AND ("precision medicine" OR "personalized medicine"):ti,ab,kw AND ("Neoplasm" OR "cancer"):ti,ab,kw NOT ("Sequence Analysis, DNA"):ti,ab,kw NOT ("next generation sequencing" OR "next-Generation Sequencing"):ti,ab,kw (Word variations have been searched)<br>#2 MeSH descriptor: [Patient Preference] explode all trees<br>#3 MeSH descriptor: [Disease Attributes] explode all trees<br>#4 MeSH descriptor: [Precision Medicine] explode all trees<br>#5 MeSH descriptor: [Precision Medicine] explode all trees<br>#6 MeSH descriptor: [Precision Medicine] explode all trees<br>#7 MeSH descriptor: [Neoplasms] explode all trees<br>#8 MeSH descriptor: [Neoplasms] explode all trees<br>#9 MeSH descriptor: [Sequence Analysis, DNA] explode all trees<br>#10 MeSH descriptor: [High-Throughput Nucleotide Sequencing] explode all trees<br>#11 MeSH descriptor: [High-Throughput Nucleotide Sequencing] explode all trees<br>#12 #2 OR #3<br>#13 #12 AND #4 AND #7 NOT #9 NOT #10<br>#14 #12 AND #4 AND #7 | 23 |

**Appendix 2.**

**Survey 1:**

If you have 2 options to choose for the cancer treatment, which one will you prefer? (please tick below box)

| **Treatment characteristics** | **Option A** | **Option B** |
| --- | --- | --- |
| Access to treatment | Not easy | Easy |
| Change in Quality of life | Increase | Increase |
| Effect on life expectancy | Between a loss of 6 months to a gain of 2.5 years, average gain of 1 year | Between a gain of 0 years and a gain of 8 years, average gain of 4 years |
| Out-of-pocket in 6 months | $1,000 | $2,000 |

Prefer Treatment option A ☐ Prefer Treatment option B ☐

| **Treatment characteristics** | **Option A** | **Option B** |
| --- | --- | --- |
| Access to treatment | Not easy | Easy |
| Change in Quality of life | Increase | No change |
| Effect on life expectancy | Between a loss of 3 months to a gain of 9 months, average gain of 6 months | Between a gain of 6 months to a gain of 1.5 years, average gain of 1 year |
| Out-of-pocket in 6 months | $1,300 | $1,300 |

Prefer Treatment option A ☐ Prefer Treatment option B ☐

| **Treatment characteristics** | **Option A** | **Option B** |
| --- | --- | --- |
| Access to treatment | Easy | Easy |
| Change in Quality of life | Increase | No change |
| Effect on life expectancy | Between a loss of 6 months to a gain of 2.5 years, average gain of 1 year | Between a gain of 2 years and a gain of 6 years, average gain of 4 years |
| Out-of-pocket in 6 months | $1,600 | $2,000 |

Prefer Treatment option A ☐ Prefer Treatment option B ☐

**Survey 2:**

If you have 2 options to choose for the cancer treatment, which one will you prefer? (please tick below box)

| Treatment characteristics | Option A | Option B |
|---|---|---|
| Access to treatment | Easy | Not easy |
| Change in Quality of life | No change | No change |
| Effect on life expectancy | Between a gain of 1 month to a gain of 5 months, average gain of 3 months | Between a loss of 3 months to a gain of 9 months, average gain of 6 months |
| Out-of-pocket in 6 months | $1,000 | $1,300 |

Prefer Treatment option A ☐ Prefer Treatment option B ☐

| Treatment characteristics | Option A | Option B |
|---|---|---|
| Access to treatment | Not Easy | Not easy |
| Change in Quality of life | No change | Increase |
| Effect on life expectancy | Between a loss of 6 months to a gain of 2.5 years, average gain of 1 year | Between a gain of 2 years and a gain of 6 years, average gain of 4 years |
| Out-of-pocket in 6 months | $1,300 | $2,000 |

Prefer Treatment option A ☐ Prefer Treatment option B ☐

| Treatment characteristics | Option A | Option B |
|---|---|---|
| Access to treatment | Not easy | Not easy |
| Change in Quality of life | Increase | Increase |
| Effect on life expectancy | Between a gain of 1 month to a gain of 5 months, average gain of 3 months | Between a gain of 0 years and a gain of 8 years, average gain of 4 years |
| Out-of-pocket in 6 months | $1,000 | $1,600 |

Prefer Treatment option A ☐ Prefer Treatment option B ☐

**Survey 3:**

If you have 2 options to choose for the cancer treatment, which one will you prefer? (please tick below box)

| **Treatment characteristics** | **Option A** | **Option B** |
|---|---|---|
| Access to treatment | Easy | Easy |
| Change in Quality of life | Increase | No change |
| Effect on life expectancy | Between a loss of 6 months to a gain of 2.5 years, average gain of 1 year | Between a gain of 1 month to a gain of 5 months, average gain of 3 months |
| Out-of-pocket in 6 months | $2,000 | $1,600 |

Prefer Treatment option A ☐ Prefer Treatment option B ☐

| **Treatment characteristics** | **Option A** | **Option B** |
|---|---|---|
| Access to treatment | Easy | Easy |
| Change in Quality of life | No change | No change |
| Effect on life expectancy | Between a loss of 3 months to a gain of 9 months, average gain of 6 months | Between a gain of 0 years and a gain of 8 years, average gain of 4 years |
| Out-of-pocket in 6 months | $1,300 | $1,000 |

Prefer Treatment option A ☐ Prefer Treatment option B ☐

| **Treatment characteristics** | **Option A** | **Option B** |
|---|---|---|
| Access to treatment | Easy | Not easy |
| Change in Quality of life | Increase | No change |
| Effect on life expectancy | Between a gain of 1 month to a gain of 5 months, average gain of 3 months | Between a gain of 2 years and a gain of 6 years, average gain of 4 years |
| Out-of-pocket in 6 months | $2,000 | $1,600 |

Prefer Treatment option A ☐ Prefer Treatment option B ☐

**Survey 4:**

If you have 2 options to choose for the cancer treatment, which one will you prefer? (please tick below box)

| **Treatment characteristics** | **Option A** | **Option B** |
|---|---|---|
| Access to treatment | Not easy | Easy |
| Change in Quality of life | Increase | No change |
| Effect on life expectancy | Between a gain of 2 years and a gain of 6 years, average gain of 4 years | Between a loss of 6 months to a gain of 2.5 years, average gain of 1 year |
| Out-of-pocket in 6 months | $1,600 | $1,000 |

Prefer Treatment option A ☐ Prefer Treatment option B ☐

| **Treatment characteristics** | **Option A** | **Option B** |
|---|---|---|
| Access to treatment | Not easy | Not easy |
| Change in Quality of life | No change | No change |
| Effect on life expectancy | Between a loss of 3 months to a gain of 9 months, average gain of 6 months | Between a gain of 6 months to a gain of 1.5 years, average gain of 1 year |
| Out-of-pocket in 6 months | $1,000 | $2,000 |

Prefer Treatment option A ☐ Prefer Treatment option B ☐

| **Treatment characteristics** | **Option A** | **Option B** |
|---|---|---|
| Access to treatment | Not easy | Easy |
| Change in Quality of life | Increase | Increase |
| Effect on life expectancy | Between a gain of 0 years and a gain of 8 years, average gain of 4 years | Between a gain of 6 months to a gain of 1.5 years, average gain of 1 year |
| Out-of-pocket in 6 months | $1,600 | $1,300 |

Prefer Treatment option A ☐ Prefer Treatment option B ☐
